# Genome sequence and analysis of *Lactobacillus helveticus*

**DOI:** 10.3389/fmicb.2012.00435

**Published:** 2013-01-11

**Authors:** Paola Cremonesi, Stefania Chessa, Bianca Castiglioni

**Affiliations:** Institute of Agricultural Biology and Biotechnology, National Research CouncilLodi, Italy

**Keywords:** *Lactobacillus helveticus*, genome sequence, IS element, pseudogenes, transporter, probiotic, ACE inhibitor activity, plasmid

## Abstract

The microbiological characterization of lactobacilli is historically well developed, but the genomic analysis is recent. Because of the widespread use of *Lactobacillus helveticus* in cheese technology, information concerning the heterogeneity in this species is accumulating rapidly. Recently, the genome of five *L. helveticus* strains was sequenced to completion and compared with other genomically characterized lactobacilli. The genomic analysis of the first sequenced strain, *L. helveticus* DPC 4571, isolated from cheese and selected for its characteristics of rapid lysis and high proteolytic activity, has revealed a plethora of genes with industrial potential including those responsible for key metabolic functions such as proteolysis, lipolysis, and cell lysis. These genes and their derived enzymes can facilitate the production of cheese and cheese derivatives with potential for use as ingredients in consumer foods. In addition, *L. helveticus* has the potential to produce peptides with a biological function, such as angiotensin converting enzyme (ACE) inhibitory activity, in fermented dairy products, demonstrating the therapeutic value of this species. A most intriguing feature of the genome of *L. helveticus* is the remarkable similarity in gene content with many intestinal lactobacilli. Comparative genomics has allowed the identification of key gene sets that facilitate a variety of lifestyles including adaptation to food matrices or the gastrointestinal tract. As genome sequence and functional genomic information continues to explode, key features of the genomes of *L. helveticus* strains continue to be discovered, answering many questions but also raising many new ones.

## GENERAL GENOME FEATURES AND HISTORY

The number of complete genome sequences is increasing rapidly, thanks to the availability of the so called next-generation sequencing (NGS) technologies, enabling the production of high-quality genome sequence data at affordable costs and with easy to use protocols. These new technologies rely on a combination of template preparation, sequencing and imaging, and genome alignment and assembly methods (Metzker, 2010) and have been successfully applied to *de novo* sequencing, whole-genome re-sequencing, transcriptomics, DNA methylation analysis, and metagenomics (MacLean et al., 2009).

The three mostly used NGS systems are the Roche Genome Sequencer FLX System (FLX), the Illumina Genome Analyzer (GA), and the Applied Biosystems SOLiD system (SOL-iD). A significant feature of NGS is that it produces millions of short sequence reads (50–400 bp), for a total amount of data varying from half to more than 100 Gbp (Miller et al., 2010) for each analysis. Advancements in sequencing chemistry, instrumentation, and software had now increased the efficiency of the different systems. To assemble millions of short sequence data to extract sequence features of DNA samples requires a great computational effort. Moreover, the assembly results may be biased by the quality of sequencing reads, such as error rate and systematic sequence bias in the obtained short reads (Shendure and Ji, 2008), therefore bioinformatics tools have been developed to overcome these problems. Although there is interest in applying Illumina’s sequencing platform to whole bacterial genome sequencing (Qin et al., 2010), one of the most used technologies is the FLX. Also the 16S rRNA gene sequencing has primarily been performed using Roche-454’s sequencing platform (FLX). 16S rRNA gene sequencing is typically applied to analyze the bacterial and archaeal species since no other molecular marker is found in all organisms, has as low a rate of horizontal gene transfer (HGT) and recombination, and gives so much genetic information to differentiate closely related organisms.

Before the use of NGS, identification and characterization of bacterial species required much work, since many specific methods (culture procedures, genetic detection, etc.) had to be applied for each of the possible species. NGS has already been used in the genome analysis of many bacterial pathogens, large-scale comparative studies, metagenomics (a culture-independent method for analyzing by sequencing with a single common protocol various microorganisms and genes present in a certain environment), and to the analysis of the so-called “probiotics” (Holt et al., 2008; Nakamura et al., 2008; MacConaill and Meyerson, 2008; Palacios et al., 2008; Monot et al., 2009; Nakamura et al., 2011; Nakaya et al., 2011), health-promoting and mucosa-adherent species, defined as “live microorganisms, which when administered in adequate amounts confer a health benefit on the host” (FAO/WHO, 2001).

Added as starter cultures or selected in naturally fermented foods, the best studied and most widely used commercial probiotic species belong to the genera *Bifidobacterium* and *Lactobacillus* (Felis and Dellaglio, 2007; Kleerebezem and Vaughan, 2009). Their health-promoting activity could be due to the production of biologically active peptides. Biopeptides, specific protein fragments with a positive effect on body functions or conditions (Kitts and Weiler, 2003), are inactive within the sequence of the parent protein and can be released by enzymatic proteolysis during gastrointestinal digestion or food processing (Fitzgerald and Murray, 2006; Korhonen and Pihlanto, 2006). Their potential action includes opioid agonist and antagonist, antihypertensive, antithrombotic, immunomodulatory, antimicrobial, and mineral transportation effects (Meisel, 1997). Moreover, the microbiota contribute to competitive exclusion, maintenance of barrier function, enhancement of a balanced microbial flora, modulation of signal transduction (Sherman et al., 2009), and lowering of blood cholesterol levels (Ataie-Jafari et al., 2009).

With increasing interest in the benefits and the applications of probiotic microbes, the Lactic Acid Bacteria Genome Consortium (LABGC) and numerous food and pharmaceutical companies have sequenced a broad range of health-promoting and industrially relevant strains (Liu et al., 2005), leading to the publication of the genomes of a number of lactobacilli (Kleerebezem et al., 2003; Pridmore et al., 2004; Altermann et al., 2005; Chaillou et al., 2005; Claesson et al., 2006; Makarova et al., 2006; van de Guchte et al., 2006). Even if *L. helveticus* is not considered a probiotic culture, certain strains are responsible for proteolytic generation of antihypertensive peptides from milk during fermentation (Takano, 2002). A delayed breast tumor growth was also demonstrated for milk fermented with *L. helveticus* R389 due to decreasing interleukin-6 (IL-6) and increasing IL-10 in serum, mammary glands, and tumor-infiltrating immune cells (Liu et al., 2010). 

There is a great interest in analyzing the genome of this species also because of the widespread use of *L. helveticus* in cheese technology. The comparison of genomic sequences of *Lactobacillus* species employed in the production of fermented milk, meat, and plant products and routinely isolated from different human organs, helps revealing the structure and function of the different microbial communities. In this way it will be easier to understand how the different species produce both undesired and highly desirable traits, such as faster ripening, reduced bitterness, or increased flavor notes (Kiernan et al., 2000; Hannon et al., 2003; Hickey et al., 2006), and to identify genes with industrial potential for the production of cheese and other milk derivatives.

The completed genome projects are now 4056, including 183 eukaryotic, 179 archaeal, and 3694 bacterial genome sequences ^1^. In particular, since the first bacterial genome (*Haemophilus influenzae*) was completely sequenced in 1995, the genomic analysis of bacterial has advanced incredibly in a short time: the available sequences were 17 by the end of 1998, 149 in 2003, and 3118 in July 2012.

## GENOMICS OF *L. helveticus*

The genus *Lactobacillus* has more than 150 cultured species ^2^ and is noteworthy for its extreme phylogenetic, phenotypic, and ecological diversity. *Lactobacillus* species are employed in the production of a wide range of fermented milk, meat, and plant products and are also routinely isolated from the vagina and the gastrointestinal tract (GIT). This broad range of environmental niches is reflected in the diversity of species belonging to the genus *Lactobacillus*. However, the real extent of *Lactobacillus* diversity is not fully known and culture-independent 16S rRNA gene surveys of complex ecosystems (e.g., the human gut microbiota) are expected to uncover novel phylotypes that belong to the genus *Lactobacillus *(Ventura et al., 2009).

The microbiological characterization of lactobacilli is historically better developed than that of *Bifidobacteria*, but the genomic analysis is recent. Of the 30 sequenced and published *Lactobacillus* genomes, eight (*Lactobacillus acidophilus*, *Lactobacillus casei*, *Lactobacillus fermentum*, *Lactobacillus gasseri*, *Lactobacillus johnsonii*, *Lactobacillus reuteri*, *Lactobacillus salivarius*, and *Lactobacillus plantarum*) are from cultures or species that are considered to be probiotic. Comparative analysis highlighted extensive loss of ancestral genes since divergence from a common ancestor and subsequent lineage-specific gene loss (Makarova et al., 2006). This loss is mainly due to the transition to a nutritionally rich environment, which allowed for a general metabolic simplification reflected in changes in sugar metabolism genes, in the loss of genes for the biosynthesis of cofactors and amino acids, and in the increase in number of transporters and peptidases genes (Callanan et al., 2008).

The genomes of five *L. helveticus* strains (**Table 1**) were sequenced to completion before September 2012 and for four of them a paper describing genome features was also published: DPC 4571 (Callanan et al., 2008), DSM 20075 (direct sequence submission), H10 (Zhao et al., 2011), MTCC 5463 (Prajapati et al., 2011), and R0052 (Tompkins et al., 2012).

**Table 1:** Genome features of the five *Lactobacillus helveticus *strains (DPC 4571, DSM 20075, H10, MTCC 5463, and R0052) sequenced to completion.

	Strain				
	DPC 4571	DSM 20075	H10		MTCC 5463	R0052	
Source	Swiss cheese	Emmental cheese	Traditional fermented milk		Vaginal tract	Sweet acidophilus milk	
GenBank	CP000517	ACLM00000000	NC_017467	NC_017468		CP003799	
Gold ID^*^	Gc00690	Gi02795	Gc01622	Gc01622	Gi08674	Gc02320	
Status	Chromosome	Chromosome	Chromosome	Plasmid	Chromosome	Chromosome	Plasmid
Size	2,080,931	1,808,667	2,145,899	26,484	1,911,350	2,129,425	6,414
GC%	37.1	36.8	36.8	36.8	36.7	36.8	
Genes	1,838	2,129	2,148	25	2,307	2,084	
Proteins	1,610	2,078	1,978	25	2,239	2,011	
rRNA	12	3	12		7		
tRNA	61	3	62		61		
Pseudogene	155		96				
Center	MWG-Biotech	BCM-HGSC	MWG-Biotech	Anand Agricultural University	Institut Rosell Lallemand Inc.	
Country	Ireland	USA	China	India	Canada	
Completion date	2007-11-15	2009-09-29	2011-02-16	2011-04-08	2012-09-05	
Sequencing depth	7.7x , not specified	33x - FLX	l43.8x - FLX 866x - GA	FLX	51x - FLX	
Reference	Callanan et al. (2008)	Direct sequence submission	Zhao et al. (2011)		Prajapati et al. (2011)	Tompkins et al. (2012)	Hagen et al. (2010)

*Sequences data are available in the public databases also for two other strains not completely sequenced (CNRZ32, H9).*

* Genomes online Database Accession number.

The strains were isolated from different environment: DPC 4571 strain was isolated from a Swiss cheese and was interesting because of its highly desirable traits in cheese manufacture (Callanan et al., 2008); DSM 20075 was isolated from Emmental cheese; H10 strain was isolated from a traditional fermented milk in Tibet, China (Airidengcaicike et al., 2010); MTCC 5463 strain was isolated from the vaginal tract of a healthy adult female in India (Khedekar et al., 1991) and showed significant antimicrobial activity (Khedekar et al., 1990), a hypocholesterolemic effect in humans (Ashar and Prajapati, 2000), and positive immunomodulating effects in a chick model (Patidar and Prajapati, 1999); R0052 was isolated from a sweet acidophilus milk, used in commercial probiotic preparation and initially identified as a *L. acid-*ophilus strain based on its phenotypic characteristics (Tompkins et al., 2012).

**FIGURE 1:**
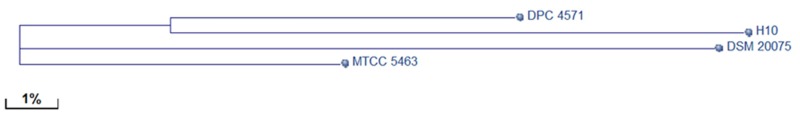
**Dendogram of *L. helveticus* strains sequenced to completion (DPC 4571, DSM 20075, H10, and MTCC 5463) based on genomic Blast**.

The *L. helveticus* complete genome sequence consisted in a circular chromosome of about 2 Mbp, with some differences among strains (**Figure 1**): 2,080,931 nucleotides with the average GC content of 37.73% for DPC 4571 (Callanan et al., 2008); 2,145,899 bp with the average GC content of 36.79% for H10 (Zhao et al., 2011); 1,911,350 bp for MTCC 5463 (Prajapati et al., 2011); 2,129,425 bp for R0052 (Tompkins et al., 2012). Also the sequencing strategy was a little different. For DPC 4571 the sequence was determined by random shotgun sequencing of a small (about 1 kb) insert library combined with a larger one (2–2.5 kb) only where required. The sequence depth of 7.7× was obtained by MWG-Biotech AG (Ebersberg, Germany; Callanan et al., 2008). For H10 it was obtained combining FLX and GA sequencing technology using respectively an insert library of about 8 and 3 kb and reaching a 43.81- and a 866-fold coverage of the genome (Zhao et al., 2011), whereas for MTCC 5463 and R0052 it was performed by only the FLX system (Prajapati et al., 2011; Tompkins et al., 2012). For the R0052 a standard library, a 3-kb- insert paired-end library, and an 8-kb-insert paired-end library were used.

The annotation of the DPC 4571 genome sequence identified 2,065 predicted ORFs (open reading frames), the 19% (388 ORFs) reclassified as large complement of 217 pseudogenes, and a large number of repeat elements. The predicted ORFs were assigned to 10 functional groups including: pseudogenes, cellular processes, transcriptional regulators, translation and ribosomial structure, metabolism, stress, lysis (also proteolysis and lipolysis), transport, DNA replication/modification/repair, and poorly/un-characterized regions. In the H10 strain 2,049 protein-coding genes, 4 rRNA operons, and 62 tRNA genes were found in the chromosome and 25 protein-coding genes in the plasmid. Compared with DPC 4571, 300 more genes were found and 130 were absent in strain H10, mostly encoding for putative uncharacterized proteins and transposases. Most of the other functional genes are conserved in H10, with some differences in the transport systems, maybe because of their different environmental adaptations (Schroeter and Klaenhammer, 2009). In the MTCC 5463 strain 2,046 coding sequence regions and 71 RNA genes (8 rRNA, 59 tRNA, and 4 5S RNA) were reported, but the subsystems structure was similar to the one of DPC4571 only for seven subsystems: photosynthesis, iron acquisition and metabolism, motility and chemotaxis, secondary metabolism, stress response, nitrogen metabolism, and dormancy and sporulation. In the remaining subsystems considerable variation were observed. Compared with DPC 4571, MTCC 5463 has 57 more genes (12 for carbohydrate utilization) in 15 major categories. In the R0052 strain 1,980 coding sequences, including 73 RNA genes, were found in the chromosome and 8 ORFs in the plasmid.

The *in silico* analyses of the biosynthetic capabilities of DPC 4571 predicted a dependency on external supplies of amino acids and cofactors, since it can synthesize only four amino acids (aspartate, asparagine, cysteine, and serine), and appears to be incapable of synthesizing most of the vitamins (e.g., thiamine, riboflavin, and vitamin B6) and cofactors necessary for its growth. Instead, the results are indicative of a diverse substrate utilization (especially carbohydrate) and processing for *L. helveticus* MTCC 5463. The presence of biotin synthesis genes and differences in cofactors, vitamins, prosthetic groups, and pigments suggest the differential ability of the strain in the production of such bioactive compounds, in contrast to *L. helveticus* DPC 4571 (Prajapati et al., 2011).

Comparing DPC 4571 and H10 strains, Zhao et al. (2011) found that there were two proteinase-encoding genes, both of which were annotated as pseudogenes in strain DPC 4571 and one of which was a pseudogene in strain H10. Strain DPC 4571 possesses three lactic acid bacteria (LAB) peptide transport systems, the oligopeptide Opp transport system, and the di-/tripeptide transport system, Dpp and DtpT (identified as encoded by pseudogenes). In contrast, strain H10 has two peptide transport systems, the Opp and dtpT systems. Twenty-six peptidase-encoding genes were present in each strain; one of these genes was identified as a pseudogene in strain DPC 4571, whereas two were identified as pseudogenes in strain H10. This indicates that the proteolytic activity may differ not only between species but also between different strains in *L. helveticus*.

Regarding nucleotide biosynthesis, the DPC 4571 has the complete pathway to generate pyrimidines *de novo* and all but one enzyme necessary for the *de novo* synthesis of purines, making the DPC 4571 auxotrophic for purines but capable of synthesizing pyrimidines *de novo*. As a cheese culture *L. helveticus* DPC 4571 is characterized by the presence of nine genes with potential lytic activity (rapid lysis of the cheese matrix) together with 24 genes (both already described and newly identified genes) with significant similarity to known peptidase genes.

## TRANSPORTER

Although a large number of transport systems appear to be lost in some LAB, transporters still make up 13–18% of their genomes, a number larger than what is found in many other bacteria. The diverse environments occupied by LAB require the ability to transport and utilize a variety of substrates in order to survive (Schroeter and Klaenhammer, 2009). The large number of transporters corresponds to the adaptation to nutrient-rich environments and subsequent loss of biosynthetic pathways. Amino acid transporters represent the largest number of uptake systems, followed by sugar, cation/anion, and peptide transporters (Lorca et al., 2007).

The lhv_1885 di-/tripeptide transporter gene of *L. helveticus* DPC 4571, frameshifted of about a quarter of the way along, is unlikely to produce a functional protein and therefore, it is annotated as a pseudogene. There is a functional oligopeptide transporter lhv_2931 that is probably fulfilling the peptide transport role, whereas lhv_1028 and lhv_1375 act as amino acid transporters (Callanan et al., 2008). *L. helveticus* has additional genes for fatty acid biosynthesis and specific amino acid metabolism, but notably fewer cell-surface proteins and phosphoenolpyruvate phosphotransferase systems for sugar utilization (Altermann et al., 2005; Callanan et al., 2008). Additionally, no transporters for complex carbohydrates, such as raffinose and fructo-oligosaccharides, are encoded by the *L. helveticus *genome, reflecting the degree of adaptation of *L. helveticus* to a milk environment.

## GASTROINTESTINAL AND PROBIOTIC-RELATED GENES

Among the environments to which the LAB have to adapt, the GIT currently pays a great deal of interest. Genes have been identified that encode for proteins involved in probiotic functions including acid/bile tolerance, surface proteins/adherence, gene transfer, and carbohydrate utilization (Klaenhammer et al., 2005), such as genes encoding for cell surface mucus-binding proteins. Although lactobacilli and other LAB make up a small portion of the total gastrointestinal microbial community, they are predominant microbiota in the small intestine and considered to play a pivotal role in its protection (Heilig et al., 2002; Hayashi et al., 2005).

Analysis of the *L. helveticus* DPC 4571 genome provided important insights into the evolution of dairy cultures and related probiotic bacteria. Half the phosphotransferase systems, cell wall-anchoring proteins, and all the mucus binding proteins predicted in probiotic *L. acidophilus* NCFM were deleted or classed as pseudogenes in DPC 4571 (Callanan et al., 2008). A consistent pattern of specialization for the milk niche has now been documented for both species. From the perspective of probiotic-culture research, the selective loss of functionally related groups of genes from the DPC 4571 genome points to the importance of those genes in the probiotic genome and the presence of selective pressure to maintain them in the gut environment.

Interestingly, *L. helveticus* that appears to have diverged from other lactobacilli via adaptation to a milk environment does not contain any mucus-binding proteins. Only in R0052, a probotical strain, genes encoding three mucus-binding protein precursors were found. These proteins are thought to play an important role in the adhesion to the intestinal mucus layer and may demonstrate that this particular strain is able to persist in the gut. Actually a total number of 83 genes shared with the *L. acidophilus* strains that were not observed in any of the other fully sequenced strains of *L. helveticus* were observed in R0052 (Tompkins et al., 2012). Moreover, *L. helveticus* DPC 4571 encodes less than half the cell wall proteins of the closely related gastrointestinal commensal *L. acidophilus* (Callanan et al., 2008). Also, α-1,6-glucosidase enzymes involved in the breakdown of oligosaccharides released from starch by amylases are more numerous in multi-niche *L. plantarum* and gut strains, with most common probiotic strains containing two copies. *L. helveticus* DPC 4571 is the only dairy strain to possess this type of enzyme (one copy; Slattery et al., 2010).

It should be also noted that although *L. helveticus* is not considered a probiotic culture, certain strains have been shown to exert beneficial effects (Takano, 2002; Tompkins et al., 2012) and it is intriguing that the mechanism is closely related to one of the commensal gastrointestinal lactobacilli. Consequently, one might expect that *L. helveticus* could have some probiotic potential when ingested orally. Bacterial infections of the GIT represent a major global health problem, even in the presence of normally effective mucosal immune mechanisms, and are important targets for vaccine development. Many probiotics have been reported to be useful in the treatment of disturbed intestinal microflora and diarrheal diseases. Vinderola et al. (2007) examined whether the production of metabolites during fermentation by *L. helveticus* R389 could confer enhanced protection against *Salmonella typhimurium* infection and whether potentially bioactive metabolites produced in fermented milk contributed to any protection observed in BALB/c mice. They observed that both the milk fermented by *L. helveticus* R389 and the non-bacterial fermented milk fraction conferred protection involving not only the probiotic bacteria but also the biological metabolites produced during fermentation of milk. It was also demonstrated that the mucosal immune response was involved in the protection observed and that it was not limited to competitive interactions between *L. helveticus*.

## PSEUDOGENES AND INSERTION SEQUENCE (IS) ELEMENTS

As evidenced by the recent and ongoing genome reduction of LAB, the presence of pseudogenes is often in relatively high numbers compared with other groups of bacteria (Schroeter and Klaenhammer, 2009). Niche adaptation occurs in a number of ways, namely gene loss or decay, lateral gene transfer or gene up regulation or mutation. This feature is particularly common in organisms that are associated with nutrient-rich food environments such as *L. bulgaricus*, *L. helveticus*, *L. lactis*, *S. thermophilus*, and *Oenococcus oeni*. In particular, *L. helveticus* DPC 4571 is reported (Callanan et al., 2008) to have a large complement of 217 pseudogenes, 36% of which had similarity to transposase enzyme genes, indicating they belonged to IS elements, a common feature recognized as a mechanism of transposition regulation. Among the 141 non-transposase-encoding pseudogenes of DPC 4571, 19 pseudogenes were predicted transport protein while 11 were as energy metabolism genes. A significant number of pseudogenes-encoded putative regulators (15 pseudogenes) and amino acid metabolism (9 pseudogenes), and a number of nucleotide metabolism genes (6 pseudogenes) also appeared to be inactivated (Callanan et al., 2008). These pseudogenes are non-functional due to frameshift, nonsense mutation and deletion or truncation. Examples of this are the bile salt hydrolase gene of *L. helveticus*, where a frameshift at nucleotide position 417 introduces a stop codon, rendering the gene inactive (O’Sullivan et al., 2009), and the di-/tripeptide transporter lhv_1885 that is frameshifted so that the protein is unlikely to be functional (Slattery et al., 2010).

IS elements are short DNA sequences (1–2 kb) capable of independent transposition within and between bacterial genomes (Mahillon and Chandler, 1998). Their capacity for independent mobility demonstrates the parasitic nature of these elements (Doolittle and Sapienza, 1980); however, they can also be regarded as having a positive influence, as they assist in promoting genetic variation (Arber, 2000). Thus, even though the primal character of these elements remains unclear in that they may be considered simply as selfish DNA elements, their impact on the architecture of microbial genomes is undeniable. They have sometimes been viewed simply as genomic parasites that are maintained only by transposition and HGT. However, microbial evolution experiments demonstrated that IS elements contribute to the generation of genetic diversity and can help promote adaptation of the microbial population (Schneider and Lenski, 2004).

Recent bioinformatic analysis carried out on the genome sequence of *L. helveticus* DPC 4571 has allowed an extraordinary number of IS elements (213 in total) to be identified (Callanan et al., 2008). A further investigation aimed at assessing the role of IS elements in the genome evolution of *L. helveticus* and suggested that their expansion is influenced by environmental factors and that there is a correlation between the types of IS elements participating in horizontal gene transfer and their copy number (Kaleta et al., 2010). To date, nineteen IS elements have been described for *L. helveticus*, namely IS1201 (Tailliez et al., 1994); ISL2 and its synonym ISL2A (Zwahlen and Mollet, 1994); ISLh1 (Pridmore et al., 1994); ISLhe1, ISLhe6, and ISLhe15 (Callanan et al., 2005); ISLhe2, ISLhe4, ISLhe9, ISLhe65, ISLhe5, ISLhe7, ISLhe12, ISLhe30, ISLhe60, ISLhe61, ISLhe63, and ISLhe66 (Callanan et al., 2008), although at present only 12 of them are included in the IS element database ^3^. A few IS elements previously found in the chromosome of other LAB species, namely *L. delbrueckii *(ISL5 and ISL7), *L. johnsonii* (ISLjo1 and ISLjo5), and *Leuconostoc mesenteroides* (IS1165), as well as in transposon Tn3692 of *L. crispatus* (IS of Tn3692) have also been identified in the DPC 4571 genome (Callanan et al., 2008). The occurrence of these IS elements in a collection of ten *L. helveticus* reference strains was further confirmed by cDNA microarrays (Kaleta et al., 2010).

Applicability of genetic methods in strain discrimination has been demonstrated in restriction analysis followed by hybridization with IS1201 element (Giraffa et al., 2000), which is the only example of IS elements employed in *L. helveticus* typing strategies to date. Analysis of the recently sequenced *L. helveticus* DPC 4571 genome reveals a remarkable abundance of IS elements (Callanan et al., 2008). Given the finding that *L. helveticus* DPC 4571 has so many IS sequences whose localization is defined in the chromosome sufficiently stable to allow to be used as markers, these could be used as a basis to distinguish between strains of *L. helveticus*.

## ACE INHIBITOR ACTIVITY

**FIGURE 2:**
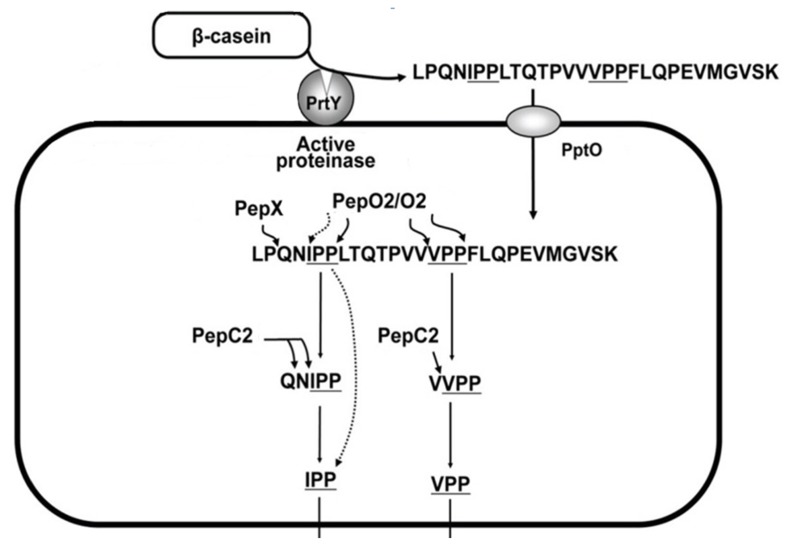
**Postulated proteolytic system for Val-Pro-Pro and Ile-Pro-Pro processing in *L. helveticus***. Modified from Wakai et al. (2012). The proteolytic action of the cell-wall proteinase (PrtY) on β-casein produces a casein peptide of 28 amino acids including VPP and IPP sequences. The peptide is incorporated into the cell by the oligopeptide transporter (PptO), and processed intracellularly to VPP and IPP by endopeptidases at the C- and N-terminal sequence. A key enzyme recently identified in *L. helveticus* CM4 strain and characterized by a homology to an endopeptidase (PepO2) can catalyzed C-terminal processing of VPPFL and IPPLT to VPP. As for the N-terminal end the release of VPP and IPP may be catalyzed by specific aminopeptidases, such as pepC2 and X-prolyl dipeptidyl aminopeptidase (PepX), since PepX is able to release the di-peptide with a sequence of X-Pro from the N-terminus and aminopeptidase may stop the hydrolysis at a X-Pro-Pro- sequence if the motif is present.

LAB possess a proteolytic system which involves the hydrolysis of milk proteins and allows the bacteria to use peptides and amino acids as nutrients (Kunji et al., 1996). Among LAB, *L. helveticus* can grow rapidly in milk because of its high proteolytic activity and resistance to acid stress (Yamamoto et al., 1994). Therefore, *L. helveticus* can release a large amount of peptides, including bioactive peptides, in fermented milk by means of proteolysis of milk proteins (Sasaki et al., 1995). These enzymes play a key role in the shortening of ripening time, in flavor development and the reduction of bitterness (Fortina et al., 1998). Recently, the contribution of *L. helveticus *cell-wall proteases to the activation of antihypertensive sequences, namely Ile-Pro-Pro (IPP) and Val-Pro-Pro (VPP) tripeptides, from the hydrolysis of casein has been demonstrated (Yamamoto et al., 1994; Scolari et al., 2002, 2006; **Figure 2**). The specific mechanism of action of these bioactive peptides, known as aceins, relies on the inhibition of angiotensin I-converting enzyme (ACE), which participates in the renin–angiotensin system, and hence, in the mediation of extracellular fluid volume, vascular resistance, and arterial blood pressure (Harrison-Bernard, 2009). The capacity of *L. helveticus* to produce antihypertensive peptides in milk-based media is strain-dependent and to date several strains (e.g., CM4, CPN4, CP790, CP611, CP615, JCM1006, JCM 1004, and LBK-16H) have been found to produce ACE-inhibitory peptides during milk fermentation (Seppo et al., 2002; Sipola et al., 2002; Aihara et al., 2005). Milk fermented by some of these strains also exhibited antihypertensive effects in animals and in clinical studies (Seppo et al., 2002; Sipola et al., 2002; Aihara et al., 2005; Jauhiainen et al., 2010). Meta-analysis for these clinical trials showed a significant reduction of blood pressure by treatment with VPP and IPP (Pripp, 2008).

Jauhiainen et al. (2010) in their study evaluated whether the milk-based drink containing IPP and VPP influence arterial stiffness, measured as augmentation index (AIx), and endothelial function in man, revealing that the long-term intake of *L. helveticus*-fermented milk containing IPP and VPP tripeptides reduces arterial stiffness in hypertensive subjects. Moreover, a current double-blind placebo controlled study revealed an improvement of arterial stiffness by oral administration of VPP and IPP for more than 8 weeks (Nakamura et al., 2011). To better understand the regulation of gene expression of the proteolytic enzymes involved in the processing of VPP and IPP by peptides and amino acids, microarray analysis for whole gene expression in the presence and absence of added peptides in the fermented milk was studied by Wakai et al. (2012). For the release of a large amount of VPP and IPP, the *L. helveticus* CM4 strain with high cell envelope-associated proteinase (CEP) activity was isolated and used in the preparation of the fermented milk product (EU Patent, 1016709A1, 1991). By DNA microarray analysis it was found that prtH2, corresponding to the CEP gene, most of the endopeptidase genes such as pepE, pepO1, pepO2, and pepO3, most of the oligopeptide transporter genes, such as dppA2, dppB, dppC, dppD, and dppF, most likely involved in the processing of VPP and IPP, were down-regulated. These results suggest that amino acids released from milk peptides in the fermented milk might down-regulate the gene expressions of some of the proteolytic enzymes and may cause repression of the release of VPP and IPP in *L. helveticus* fermented milk.

## PLASMIDS

In specific environmental niches, particularly dairy, plasmids are undoubtedly of significant importance. Plasmids, which are omnipresent in LAB, often encode for genes with technologically important traits involved in lactose/galactose utilization, proteolysis, oligopeptide transport, bacteriophage resistance, citrate utilization, bacteriocin production, and stress response (O’Sullivan et al., 2009). Plasmids are extrachromosomal elements which can replicate autonomously from the cellular chromosome of their host bacterium. *Lactobacillus* plasmids were first identified in *L. casei* by Chassy et al. (1976) and rolling circle and theta-replicating plasmids have since been observed in many species of lactobacilli. Rolling circle replicating plasmids in *L. helveticus* have been observed in a variety of strains, and have been characterized from strains SBT2161 (plasmid LJ1; Takiguchi et al., 1989), ATCC 15009T (pLH1, pLH2, and pLH3; Fortina et al., 1993) LBL4 (pLH4; Pridmore et al., 1994), S36.2 (pLJH1; de Rossi et al., 1989), and CP53 (plasmid pCP53; Yamamoto and Takano, 1996). In 1999, the complete sequence of plasmid pLH1 was obtained revealing that it was the first plasmid observed in *L. helveticus* to replicate via the theta mechanism (Thompson et al., 2001). Plasmids pLH2, pLH3, and pLH4 have been shown to be rolling circle replicating plasmids, as the replication protein genes are similar to those in the Lactococcal rolling circle replicating plasmid pWV01 (Pridmore et al., 1994).

Few phenotypic characteristics have been associated with *L. helveticus* plasmids to date. de Rossi et al. (1989) observed a drop in proteolytic activity when plasmid pLHJ1 was cured from *L. helveticus* S36.2. Fortina and Silva (1996) observed two *L. helveticus* plasmids in strain ILC 54 to be associated with lactate production and proteolytic activity and de Los Reyes-Gavilan et al. (1990) found a restriction/modification system associated with a plasmid from *L. helveticus* CNRZ1094. In 2006, Ricci et al. (2006) performed a survey of the plasmid content from *L. helveticus* cheese isolates. Three subtypes of plasmids were observed: those similar in synteny to pLH3 and with Rep proteins homologous to that of pLH3; those homologous to pLH2; and plasmid pLHp1 in a Provolone cheese isolate which appeared quite different from the previously characterized plasmids confirming the previous observation that an intergenic region between ORFs 1 and 2 of pLH2 is frequently conserved among small *L. helveticus* plasmids.

Few attempts have been made to use small plasmids from *L. helveticus* as cloning vectors. Some preliminary experiments detailing the electro-transformation of *L. helveticus* strains with plasmid pCP53 have been performed (Yamamoto and Takano, 1996), although the plasmid has not been used to introduce novel genes into *L. helveticus*. Hashiba et al. (1992) used a 1.5 kb fragment of plasmid pLJ1 (containing the plasmid’s only gene) to generate a shuttle vector capable of replication in *Escherichia coli* and *L. helveticus*. Thompson et al. (2001) were able to transfer several genes in various *L. helveticus* strains through mobilization with a pIP501-derived vector, although they were not stable during repeated sub-culturing.

The plasmid contained in *L. helveticus* R0052 comprised of eight ORFs, four of which encode proteins of unknown function. Hagen et al. (2010) identified any potential antibiotic resistance genes in the plasmid and investigated the plasmid’s origin of replication, maintenance systems, and metabolic genes revealing that pIR52-1 is a member of the recently described RepA_N family of Gram-positive theta-replicating plasmids. The repA gene of pIR52-1 is the minimal origin of replication for *L. helveticus* and other *Lactobacillus* hosts. Additionally, pIR52-1 belongs to a subgroup of the RepA_N plasmid family which have RepA proteins of high amino acid identity and a conserved, non-coding element upstream of repA which, in pIR52-1, is responsible for the control of plasmid copy number and contributes to plasmid maintenance.

## PROPHAGE

Bacteriophages present a significant challenge in industrial fermentations using LAB. Phage and phage remnants are found in the genomes of most LAB and play a prominent role in species-to-species and strain-to-strain variability. Prophage and remnants can also encode genes directing phenotypes important for host survival or functions. Although the function of these structures is not fully understood, the spacer regions share significant homology with foreign DNA elements. It is suspected that these regions are involved in protecting the host from invasion by potentially harmful foreign DNA, including that from bacteriophages and plasmids. Anyway the genome of DPC 4571 contains no complete prophage but some of its features provide acquired resistance to bacteriophages (Callanan et al., 2008).

## COMPARATIVE GENOMICS WITH OTHER LACTOBACILLI

As already described previously, more than 150 species are assigned to the genus *Lactobacillus*, subdivided into groups, even if phylogenetic relationships in the genus *Lactobacillus* have been hotly disputed. The recognized *Lactobacillus* species have traditionally been inferred using alignments of 16S rRNA gene sequences, which is the most common single-gene phylogenetic marker employed for prokaryotes. The overall phylogenetic structure of the rRNA tree in the genus *Lactobacillus *is quite complicated (Dellaglio and Felis, 2005; Canchaya et al., 2006) and often conflicts with the older, phenotypic classification (Vandamme et al., 1996). The main discrepancy in the taxonomy of the genus *Lactobacillus* is the non-correlation between phylogeny and metabolic properties (Canchaya et al., 2006). For example, there are uncertainties about the interspecies affinities within the “acidophilus complex” (Claesson et al., 2008) that consists of five species: *L. gasseri*, *L. johnsonii*, *L. acidophilus*, *L. helveticus*, and *L. delbrueckii*. In particular, the divergence between *L. gasseri/L. johnsonii*, *L. acidophilus/*L. helveticus, and *L. delbrueckii* remains unresolved. Moreover, the phylogenetic analysis of ribosomal protein sequences derived from lactobacilli and streptococci classified *L. helveticus* in the same group along with both GIT and dairy-specific species (O’Sullivan et al., 2009).

As a result of analyses of 16S rRNA gene, a few nuclear genes (Makarova and Koonin, 2007; Liu et al., 2008; Cai et al., 2009; Ventura et al., 2009) and 32 ribosomal proteins (Callanan et al., 2008), *L. delbrueckii* was found to be more closely associated with *L. acidophilus/L. helveticus* than with *L. gasseri/L. johnsonii*. When 12 genomes of the recognized species (Euzeby, 1997) have been fully sequenced, *Lactobacillus* spp. have been targeted for several comparative whole-genome analyses and large regions of synteny were observed among *L. acidophilus*, *L. gasseri*, *L. delbrueckii*, and *L. helveticus* (Altermann et al., 2005).

Afterward, 141 core protein genes were extracted from these 12 *Lactobacillus* spp. genomes to investigate the case for a single congruent genus phylogeny (Claesson et al., 2008). The study highlighted the extreme diversity across the genus *Lactobacillus*: of the four identified subgeneric groups, only the one corresponding to the “*L. acidophilus* complex” is large and well-defined enough to distinguish it clearly from the others. This study suggested also that *L. delbrueckii* diverged earliest within the “acidophilus complex,” while *L. acidophilus/L. helveticus* and *L. gasseri/L. johnsonii* clustered into another group.

In the later study of Kant et al. (2010) a larger than the aforementioned gene set (Claesson et al., 2008) of shared orthologous genes was used. It comprised 383 genes calculated analyzing 20 complete *Lactobacillus* genome. This genes set was used for the construction of a phylogenetic tree, differing slightly from the well-known 16S rRNA-based grouping and revealing the presence of three distinct and large clusters of lactobacilli. These clusters were named after the strain designation of the largest or most well-known genome they contained as: the cluster NCFM, containing *L. acidophilus *NCFM, *L. helveticus *DPC 4571, *L. gasseri *ATCC 33323, *L. crispatus *ST1, *L. johnsonii *FI9785, *L. johnsonii *NCC 533, *L. delbrueckii *ssp. *bulgaricus* ATCC BAA 365, and *L. delbrueckii *ssp. *bulgaricus *ATCC 11842; the cluster WCFS, containing *L. plantarum *WCFS1, *L. brevis *ATCC 367, *L. plantarum *JDM1, *L. fermentum *IFO 3956, *L. reuteri *DSM 20016, *L. reuteri *JCM 1112, and *L. salivarius *UCC118; the cluster GG with *L. rhamnosus *GG, *L. rhamnosus *Lc705, *L. casei *ATCC 334, *L. casei *BL23, and *L. sakei *23k. The NCFM cluster, that contains *L. helveticus* DCP4571, is not only the largest but also the most coherent. In contrast, the WCFS and GG clusters contain each an outgroup genome, that of *L. salivarius *and *L. sakei*, respectively. Although the group-specific ORFs appeared to be of specific value in defining the different genomic groups and providing insight in the origin and function of the species they include, it was not possible to identify any niche-specific genes when considering the source of the isolated strains, as previously reported for the analysis of a smaller set of genomes (O’Sullivan et al., 2009). All the nine niche-specific genes identified in that study were found to be present in other niches as well based on the present set of *Lactobacillus* genomes. Also in the more recent paper of Lukjancenko et al. (2012), where six bacterial genera containing species commonly used as probiotics for human consumption or starter cultures for food fermentation were compared, the findings could not identify common signatures, in terms of gene content.

More recently, phylogenetic relationships among LAB species were performed based on 232 orthologous genes from 28 LAB genome sequences representing all genera from four families (Zhang et al., 2011). The concatenation of all these genes allowed the recovery of a strongly supported phylogeny, providing a maximum and decisive resolution of the relationships among the LAB species examined, divided into two groups. Group 1 included families *Enterococcaceae* and *Streptococcaceae*. Group 2 included families *Lactobacillaceae* and *Leuconostocaceae*. Within group 2, the LAB species were divided into two clades. One clade comprised of the “acidophilus complex” of genus *Lactobacillus* and two other species, *L. sakei* and *L. casei*. In the acidophilus complex, *L. delbrueckii* separated first, while *L. acidophilus*/*L. helveticus* and *L. gasseri*/*L. johnsonii* were clustered into a sister group. The other clade within group 2 consisted of the salivarius subgroup, including five species, *L. salivarius*, *L. plantarum*, *L. brevis*, *L. reuteri*, *L. fermentum*, and the genera *Pediococcus*, *Oenococcus*, and *Leuconostoc*. In this clade, *L. salivarius* was positioned most basally, followed by two clusters, one corresponding to *L. plantarum*/*L. brevis* pair and *Pediococcus*, and the other including *Oenococcus*/*Leuconostoc* pair and *L. reuteri*/*L. fermentum* pair. This work revealed the phylogenetic utility of several genes, such as those relating to translation, ribosomal structure, and biogenesis (TRSB) function and a three-gene set consisting of uvrB, polC, and pbpB. These genes may be better indicators for LAB phylogenetic studies than the other subsets of genes. This study also demonstrated the occurrence of multiple, independent adaptation events in LAB species, resulting in their occupation of various habitats.

The increasing availability of LAB genome sequence data provides a good opportunity to understand the evolutionary history of LAB species. For example, the genome comparisons among 20 LAB has demonstrated that loss and decay of ancestral genes has played a key role in the evolution of Lactobacillales. Lactobacillales diverged from their *Bacillus *ancestor with an estimated loss of 600–1,200 genes from a total gene repertoire of 2,100–2,200 (Makarova and Koonin, 2007). Many of these genes encoded biosynthetic enzymes or functioned in sporulation. However, in addition to major gene losses, gene gains also occurred that seem to reflect the nutrient-rich niches, such as milk and the GIT, that are occupied by LAB. For example, genes encoding peptidases and amino acid transport proteins as well as genes involved in the metabolism and transport of carbohydrates have been duplicated. In addition, comparative analysis between GIT-associated species *L. acidophilus*, *L. gasseri*, and *L. johnsonii* and the dairy species *L. bulgaricus* and *L. helveticus* revealed that selective pressure from niche-specific adaptation has impacted on the genome evolution of these species (Makarova et al., 2006; van de Guchte et al., 2006; Callanan et al., 2008). In addition to gene duplication, HGT is also evident in probiotic lactobacilli. For example, genes encoding cell-surface factors in *L. johnsonii* and the exopolysaccharide cluster in the *L. acidophilus* complex are examples of HGT in probiotic lactobacilli (Pridmore et al., 2004; Altermann et al., 2005).

Therefore, from the perspective of microbial genome architecture, the three mechanisms recognized to generate genetic variability are point mutations, HGT, and intragenomic rearrangements. Among these mechanisms, comparisons of the completely sequenced lactobacilli genomes revealed that the main force that drives evolution in these genomes is HGT. Even the results of genomic analysis of *L. helveticus* suggested that two major events have occurred in the diversification process of *L. helveticus* from a common ancestor with *L. acidophilus*: selective gene loss and acquisition of a large number of IS elements (Callanan et al., 2008).

**FIGURE 3:**
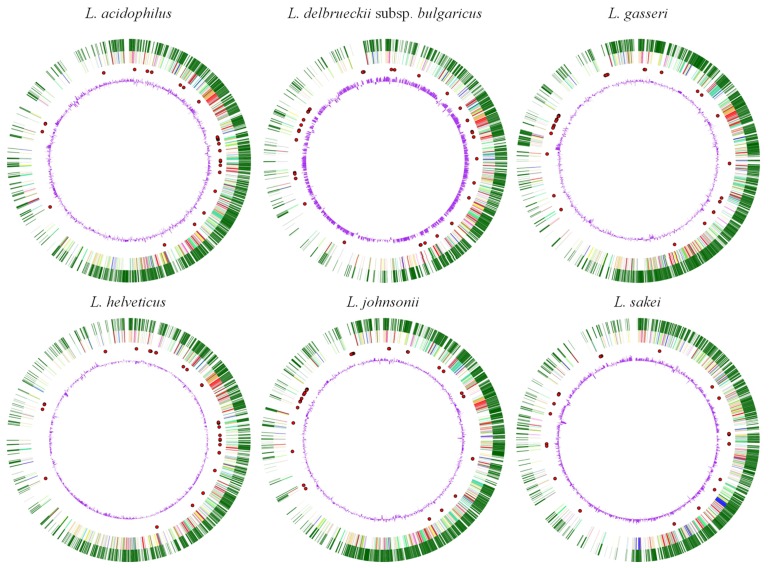
**Image of the six *Lactobacillus* species compared (*L. acidophilus* NCFM, *L. delbrueckii* subsp. *bulgaricus* ATCC11842, *L. gasseri* ATCC 33323, *L. helveticus* DPC 4571, *L. johnsonii* NCC533, *L. sakei* 23 K) generated by Microbial Genome Viewer.** From outside to inside: genes, COG categories, tRNA, GC%.

Nonetheless, the comparative analysis of the 16S rRNA of *L. helveticus* DPC 4571 revealed 98.4% identity with *L. acidophilus* NCFM, indicating that this probiotic strain was closely related to strain DPC 4571, despite the different environments these two lactobacilli inhabit (Callanan et al., 2008). From 65–75% of the genes were conserved between *L. helveticus* DPC 4571 and *L. acidophilus* NCFM. This level of chromosomal synteny is very surprising, especially since the first strain contains 213 IS elements and latter only 17; this observation suggested that it is possible for two related genomes as distinct as those of NCFM and DPC 4571 to evolve without generating intragenomic rearrangements (**Figure 3**).

As showed in **Table 2**, the NCFM and DPC 4571 genomes include basically the same set of peptidase and lysis genes, although there have been significant and possibly important changes in the peptidase protein sequences. In contrast, a definite bias toward the loss of functions associated with probiotic functionality in NCFM was observed. Half the phosphotransferase systems, cell wall-anchoring proteins, and all the mucus-binding proteins predicted in NCFM were deleted or classed as pseudogenes in DPC 4571. The *L. helveticus* genome sequence confirms that the previously reported selective loss of membrane protein and sugar metabolism genes from the *S. thermophilus* dairy culture genome (Bolotin et al., 2004) also holds for *Lactobacillus*. A consistent pattern of specialization for the milk niche has now been documented for both species. From the perspective of probiotic-culture research, the selective loss of functionally related groups of genes from the DPC 4571 genome and the maintenance in R0052 of mucus-binding genes points to the importance of those genes in the probiotic genome and the presence of selective pressure to maintain them in the gut environment. Moreover, the IS-loaded DPC 4571 genome demonstrates exceptional stability, since IS elements are generally viewed as facilitators of increased genomic rearrangement, conferring an advantage in variant generation. Overall, the DPC 4571 IS elements appear to be particularly unobtrusive considering their prominence in the genome.

**Table 2:** Comparison between the genomes of *L. helveticus* DPC 4571 and others *Lactobacilli* (LAB).

Organism	*L.helveticus* DPC 4571	*L.acidophilus* NCFM	*L.johnsonii* NCC533	*L.sakei* 23 K	*L.gasseri* ATCC 33323	*L.delbrueckii* subsp.* bulgaricus* ATCC11842
Source	Cheese	Infant feces	Human feces	Meat	Human gut	Yogurt
Size (Mb)	2.1	2	2	2	1.9	1.9
ORFs	2.065	1.864				
Pseudogenes	217	0	0	30	48	533
IS elements (Mb)	100.5	8.5	7	6		31.1
Genes	1,618	1,864	1,821	1,884	1,898	1,562
Peptidases ’lysis genes	24/9	23/9				

Considering the extraordinary abundance of IS elements in the *L. helveticus* DPC 4571 chromosome, it is noteworthy that very few ORFs are directly affected by their presence. Presumably, the vast majority of insertion events proved detrimental to some aspect of the strain’s competitiveness and so were not selected in the ensuing population. Comparative analysis described from Callanan et al. (2008) also indicated that the IS elements were not the primary agents of niche adaptation for the *L. helveticus *genome. A clear bias toward the loss of genes reported to be important for gut colonization was observed for the cheese culture, but there was no clear evidence of IS-associated gene deletion and decay for the majority of genes lost. Furthermore, an extraordinary level of sequence diversity exists between copies of certain IS elements in the DPC 4571 genome, indicating they may represent an ancient component of the *L. helveticus *genome.

The abundance of IS elements in *L. helveticus* makes it a very suitable system in which to study the role of IS elements in the evolution of bacterial genomes, particularly in ecosystems which impose challenging selective pressures. To assess this diversity and examine the level of genome plasticity within the *L. helveticus *species, an array-based comparative genome hybridization (aCGH) experiment was performed, in which 10 strains were analyzed (Kaleta et al., 2010). *L. helveticus *DPC 4571 was used as the reference strain, the other *L. helveticus *strains analyzed were DPC5607, DPC5389, DPC5367, DPC5365, DPC5360, DPC5394, DPC5352, DPC5364, and DPC1132. Results from this study suggested that IS elements do play an important role in genomic rearrangements among *L. helveticus* strains. Interestingly, even though IS elements are very abundant within the genome of *L. helveticus*, comparison of the 10 strain genomes did not reveal any deletion of large gene sets that could be expected in IS-rich chromosomes. However, variations in genetic content were detected in a number of single genes or short clusters of ORFs. Interestingly, the majority of these clusters were localized between IS elements, therefore the acquisition of IS elements might have been advantageous in terms of chromosomal rearrangement. Four of these ORFs are associated with restriction/modification which may have played a role in accelerated evolution of strains in a commercially intensive ecosystem undoubtedly challenged through successive phage attack. These findings contribute to the overall viewpoint of the versatile character of IS elements and the role they may play in bacterial genome plasticity.

Also the metabolic diversity of the *Lactobacillus *genome sequences is now available. Intestinal lactobacilli compensate for their auxotrophy by encoding multiple genes for transporters. Their genomes also contain genes that encode acid and bile resistance, capacity for uptake of macromolecules, metabolism of complex carbohydrates, and cell-surface proteins that interact with the intestinal mucosa (Pfeiler and Klaenhammer, 2007). The adaptation to life in the GIT becomes evident when the genome sequences of intestinal isolates are compared with food-adapted lactobacilli such as *L. bulgaricus* and *L. helveticus*. *L. bulgaricus* is widely used as a starter culture in yogurt fermentations and has undergone genome decay to adapt to the milk environment (van de Guchte et al., 2006). Thus, it harbors numerous degraded or partial carbohydrate pathways and bile salt hydrolase pseudogenes. In addition, *L. bulgaricus* has a preference for growth on lactose, further emphasizing its niche adaptation to milk. Compared to the closely related *L. acidophilus*, *L. helveticus* has additional genes for fatty acid biosynthesis and specific amino acid metabolism, but notably fewer cell-surface proteins and phosphoenolpyruvate phosphotransferase systems for sugar utilization (Altermann et al., 2005; Callanan et al., 2008). Additionally, few functional mucus-binding proteins or transporters for complex carbohydrates, such as raffinose and fructo-oligosaccharides, are encoded by the *L. helveticus* genome, reflecting the degree of adaptation of *L. helveticus* to a milk environment. By contrast, *L. acidophilus* has adapted to the gut ecological niche by retaining the functional gene sets that lack in *L. helveticus*, emphasizing the importance of these gene sets for probiotic functionality and niche adaptation by autochthonous lactobacilli that naturally reside in the GIT.

*Lactobacillus helveticus* is recognized among LAB for its proteolytic activity and rich assortment of proteolytic enzymes (Savijoki et al., 2006). Interest in the proteolytic system of this bacterium is tied to its ability to decrease bitterness and accelerate flavor development in cheese (Ardo and Pettersson, 1988; Drake et al., 1996) and to release bioactive peptides in milk-based foods (Laffineur et al., 1996; LeBlanc et al., 2002; Hayes et al., 2007; Gobbetti et al., 2010). However, these attributes are strain-specific, and more detailed knowledge of the proteolytic enzyme systems and amino acid catabolism in *L. helveticus* is needed to better use these organisms for food and health. Comparative genome hybridizations to explore the distribution of genes encoding such enzymes across a bank of 38 *L. helveticus *strains were performed, showing that genes for peptidases and amino acid metabolism were highly conserved across the species, whereas those for CEP varied widely (Broadbent et al., 2011). The results of this work suggested that strain heterogeneity in peptidase activity or amino acid metabolism are not based on differences in gene content, but rather are more likely due to a combination of nonsense mutations plus sequence polymorphisms that affect the expression level, specificity, or activity of the individual enzymes involved in these reactions. In contrast, marked genetic differences were discovered in the distribution of CEP paralogs, and those differences are probably very important determinants of strain functionality in cheese and in production of bioactive peptides in fermented milks.

## CONCLUSION

Genomics of *L. helveticus* and comparative genomics with other intestinal lactobacilli show a remarkable similarity in gene content despite the ecological and phenotypic diversity, and simultaneously highlighted the differences, also among strains, due to the adaptation to the different environments.

In recent years genomics has accelerated research into pathogenic, probiotic, and industrially relevant bacteria, expanding the knowledge about their evolution and genetic diversity and in some cases has elucidated the molecular basis for their different functions. For instance, the validity of probiotics can be sustained by the characterization of the specific molecular mechanisms by which these probiotic microbes elicit health benefits. The integration with functional genomics and analyses of animal and human gene expression, together with the analysis of pathogens’ genomes and interaction host–microbiota during the course of infectious diseases, can allow the understanding of the intimate microbe–microbe and host–microbe interactions.

In the same way the new omics technologies, thanks to the simultaneous analysis of great numbers of genes and proteins of any bacteria, will lead to further the understanding of the mechanisms undergoing the production of both undesired and highly desirable traits in cheese production, such as faster ripening, reduced bitterness, or increased flavor notes. The identification of new genes with industrial potential for the production of cheese and cheese derivatives will provide researchers and industry the ability to genetically tailor starter cultures to meet the needs for specific applications.

## Conflict of Interest Statement

The authors declare that the research was conducted in the absence of any commercial or financial relationships that could be construed as a potential conflict of interest.

## Footnotes

1 http://www.genomesonline.org (Accessed December 27, 2012)

2 http://www.bacterio.cict.fr/l/lactobacillus.html

3 http://www-is.biotoul.fr/is.html
